# A Comparative Evaluation of Microleakage of Two Low-Shrinkage Composites with a Conventional Resin Composite: an *In Vitro* Assessment

**Published:** 2016-03

**Authors:** Maryam Tavangar, Reza Tayefeh Davalloo, Farideh Darabi, Mahsa Karambin, Reza Kazemi

**Affiliations:** 1 Dept. of Operative and Esthetic Dentistry, Faculty of Dentistry, Guilan University of Medical Sciences, Rasht, Iran.; 2 Dentist, Rasht, Iran.; 3 Dept. of Reconstructive Sciences, University of Connecticut Health Center, School of Dental Medicine, Farmington, USA.

**Keywords:** Resin Composites, Dental Bonding, Microleakage

## Abstract

**Statement of the Problem:**

Polymerization shrinkage stress in composite restorations may lead to microleakage. Clinical methods such as using low-shrinkage composites have been suggested to overcome this problem; however, there are controversies about their efficiency in decreasing the microleakage.

**Purpose:**

This *in vitro* study was conducted to compare the microleakage of two low-shrinkage resin composites with a conventional one.

**Materials and Method:**

Fifty class V cavities of 2.5×3×2 mm (depth× length× width) were prepared in the buccal surfaces of intact bovine incisor teeth with the incisal margin on the enamel and gingival margin on the cementum. The teeth were randomly divided into 5 groups. In group 1, Clearfil APX (conventional) with SE Bond was used in 2 layers (Kuraray; Japan). In group 2, GC Kalore (low –shrinkage) with GC UniFil Bond was applied in one layer (GC Company). In group 3, the material of group 2 was applied in two layers. In group 4, FiltekP90 (low –shrinkage) with P90 System adhesive was applied in one layer (3M ESPE). In group 5, the materials of group 4 were applied in two layers. The samples were thermocycled and immersed in 0.5% fuchsin solution for 24h. The restorations were sectioned in buccolingual direction. Then they were evaluated for microleakage by using a stereomicroscope and scored as 0, 1, 2, and 3 and then Kruskal-Wallis test was used (*p*< 0.05).

**Results:**

The groups were not significantly different regarding the microleakage in the coronal and cervical margins (*p*< 0.423 and *p*< 0.212, respectively); however, the Filtek P90 yielded the best results. In all groups, except group 5 (*p*= 0.018), the cervical margins had greater microleakage than the coronal margins.

**Conclusion:**

The results suggested that low-shrinkage resin composites may not reduce the marginal microleakage. The proper use of conventional resin composites may offer comparable clinical results.

## Introduction

Although resin composites have become one of the clinicians’ primary materials of choice for most restorations in recent years, their polymerization contraction is still a fundamental problem. The polymerization contraction stresses can result in debonding at composite/tooth interface over time.[1] This conflict may also lead to enamel fracture and deflection of cusps.[1-2]

Other problems associated with the polymerization contraction of resin composite are postoperative sensitivity, microleakage, marginal staining, and eventually recurrent caries.[1] Compromising the integrity of tooth-restoration interface mayincrease the likelihood of mechanical failure.[2]

Among the several clinical approaches to reduce this stresses is using low-modulus liners between the tooth and composite which leads to uniform distribution of polymerization stress. Using low-intensity light at the beginning of polymerization can reduce the polymerization speed, hence, the composite is free to flow and less tensile stress is generated. The polymerization shrinkage stresses can also be decreased by applying incremental method for restoration. Reducing the cavity configuration factor (C-factor) is another effective alternative, considering that internal stress increases with higher C-factor.[1, 3]

The clinical application of these techniques is not only time-consuming, but the reports regarding their advantages are also controversial.[4-6] Beyond the scope of clinical restorative techniques, there are other approaches to reduce the polymerization shrinkage of resin composites such as increasing the filler loading and molecular weight in reactive groups as well as modifying the material formulation. But, in spite of these challenges, polymerization shrinkage has remained an intrinsic property of the resin matrix. Therefore, a modification in resin matrix formulation seems to be the answer to the problem.[7-8]

**Figure 1:**
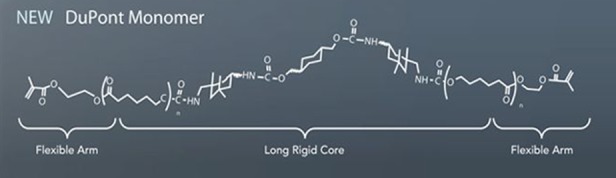
DX-511 monomer

Silorane monomer in Filtek composite (3M ESPE) and DuPont monomer in Kalore composite (GC Company) are examples of newly-marketed resin composite materials. GC Kalore is a kind of low-shrinkage composite with DuPont monomer. It is a methacrylate-base monomer with DX-511 molecule. (Figure 1)

Some scientific investigations reported that the silorane-based composites exhibited significantly lower polymerization shrinkage than the conventional methacrylate-based composites[1, 2, 4, 6-7] however; others found no significant difference between the two systems.[3, 5, 8]

 Due to the noticeable controversies, the objective of the present study was to compare the microleakage of two available low-shrinkage composites, GC Kalore (GC) and Filtek Silorane (3M-ESPE), with a conventional resin composite, Clearfil APX (Kuraray). The null hypothesis stated these two low-shrinkage composites and the conventional composite do not have statistically significant difference in terms of marginal microleakage. 

## Materials and Method

Fifty extracted intact bovine maxillary incisors, all caries-free and without crack (examined with a light curing-unit), were selected for the study. The tissue debris was removed by using a scaler, the samples were rinsed with water, and then the teeth were kept in normal saline at room temperature until used.

All the teeth were cleaned with Pumice and distilled water. Class V cavities were prepared in labial surfaces by using a high-speed handpiece and diamond fissure burs (008–Diaswiss, Swiss) under water coolant. Each bur was replaced after five preparations. The entire experiments were performed by the same operator.

The cavities all had similar dimensions (2.5×3×2 mm) as depth×length×width; so that the margins did not pass the mesial and distal line angles. The incisal margins were located 1 mm above the cement enamel junction (CEJ) and gingival margins of the cavity were terminated 1 mm below the CEJ. The limits were initially pencil-marked on the labial surfaces of the teeth. The prepared teeth were randomly divided into 5 groups (n= 10), each to be treated with specific material (Table 1).

**Table 1:** The list of tested bonding systems and resin composites

**Brand Name**	** Specific Monomer **	** Classification **	**Manufacturer**
Clearfil APX Composite	Bis-GMA Bis-EMA TEG-DMA	Methacrylate-Based	Kuraray America (New York)
Clearfil SE Bond	Methacrylate	two-step self-etch	Kuraray (Japan)
Filtek™ P90 Composite	Silorane	Low-shrinkage	3M ESPE (USA)
Filtek™ P90 Adhesive	Silorane System Adhesive	two-step self-etch	3M ESPE AG (Germany)
GC Kalore Composite	DuPont(DX-511), UDMA	Low-shrinkage	GC (Japan)
GC UniFil Bond	4-MET Adhesive Monomer	two-step self-etch	GC (Japan)

In group 1, SE Bond (self-etch system) + Clearfil APX composite (Kuraray Company) was used, and the restoration was completed with two layers of composites. In group 2, GC UniFil Bond (self-etch system) + GC Kalore composite (GC Company) was used, and the restorations were completed with two layers of composites. GC-Kalore composite is a low-shrinkage composite with DuPont monomer base. In group 3, GC UniFil Bond + GC Kalore composite (GC) was applied, and the restorations were completed with one layer of composite. In group 4, P90 adhesive system (self-etch system) + Filtek P90 composite (3M ESPE) was employed and the restorations were completed with one layer of composite. In group 5, P90 adhesive system+ Filtek P90 (3M ESPE) was used and the restorations were prepared with two layers of composites. 

In all groups, the enamel margins of cavities were etched with DenFil Etchant–37 (37% phosphoric acid gel; Vericom Co., Ltd., Korea) for 15 seconds, and then rinsed and air-dried. The A2 composite shade was used to restore all preparations.

The restoration procedures in the five groups were performed with small differences. In group 1, two-bottle self-etch SE Bond System was applied according to the manufacturer's recommendations. Self-etch primer was first applied on the walls and margins of all prepared cavities for 30 seconds and air-dried. Then, bonding was applied, air-dried, and finally light-cured for 10 seconds by using Litex 680A unit (Dentamerica; USA). To restore the cavities, Clearfil APX was applied incrementally in horizontal layers (1mm in the first layer and 1.5 mm in the second layer); each layer was light-cured for 40 seconds. In group 2, two-bottle self-etch GC UniFil Bond System and GC-Kalore composite were applied. The procedures were the same as done in group 1. In group 3, the procedures were similar to group 2, except for the cavity preparations which were restored with one layer and the entire bulk was cured for 40 seconds. In group 4, two-bottle silorane-based self-etch system (P90 Bond System) was applied according to the manufacturer’s instructions. First, the walls and margins of all cavities were conditioned by self-etch primer of P90 bonding system, air-dried, and light-cured for 10 seconds. Then, the bonding was applied, air-dried, and light-cured for 10 seconds. Next, the silorane-based Filtek P90 composite was applied. The cavity preparations were restored with one increment (bulk technique) and cured for 40 seconds. In group 5, the procedures were similar to group 4, except for the cavity preparations which were filled with two increments of composite (1 mm in the first layer and 1.5 mm in the second one); each layer was cured for 40 seconds. 

Having been restored, all samples were kept in distilled water at room temperature for 24 hours. Then the restoration surfaces were finished by using high-speed handpiece and flame-shaped finishing burs (Diaswiss, Swiss) under water coolant. The procedures were all performed by the same operator. Afterwards, the teeth were subjected to 500 thermal cycles at 5-55°C with a 30-seconds dwell time and 15-seconds transfer time. The apices were sealed with resin-based glass ionomer (GC Fuji Plus, GC Company) and light-cured for 40 seconds. The entire surfaces of the teeth were coated with two layers of nail varnish, except the restorations surfaces and 1 mm around the restoration margins.

For microleakage evaluation, the prepared specimens were immersed in 0.5 % fuchsin solution for 24 hours, then washed with water and dried. To reduce the size of samples, apical 1/3 of the roots and coronal 1/3 of crowns were removed by cylindrical-shaped bur (Diaswiss, Swiss). The specimens were then placed in plastic cube molds of 2×2×1.5 cm filled with clear polyester materials The samples were mounted in polyester to facilitate the sectioning. The samples were assigned a number (1 to 5 printed on mesial and distal sides) corresponding to one of the five designated groups. For buccolingual section of each sample, a line was drawn with a graphic pen passing over the mesiodistal midline of the restoration. Each sample was split into two halves by using a disk (Resista Omegna 68, Italy) under water coolant.

**Figure 2:**
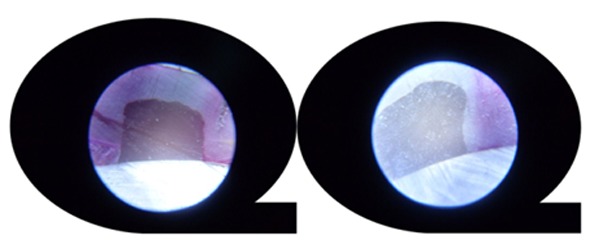
Score 3 of microleakage (left) and score 0 (right)

The prepared sections were examined under a stereomicroscope (Olympus SZX2-TR 30, Japan) at 30× magnification. The dye penetration in occlusal and gingival margins of each sample was scored between 0-3. Score 0 indicated no dye penetration at all, score 1 represented penetration up to half of the axial depth, score 2 showed penetration more than half of the axial depth without involving the axial wall, and score 3 indicated dye penetration involving the axial wall. Each half was observed twice and the greater score of dye penetration was recorded for that margin. (Figure 2)

The scores were subjected to statistical analysis by using the Fisher’s exact test and non-parametric Kruskal-Wallis analysis of variance. 

## Results

According to the results of Kruskal-Wallis test, marginal microleakage in coronal and cervical margins was not statistically different (*p*< 0.423 and *p*< 0.212, respectively). Data analysis represented that coronal/occlusal margins of 26 samples (%52) and cervical margins of 9 samples (%18) did not have any evidence of dye penetration. The highest and lowest percentage of score 0 (no dye penetration) in the coronal margin was observed, respectively, in group 1 (Clearfil APX composite) and group 2 (double-layer Kalore composite). In coronal margin, the highest percentage of dye penetration with involving axial wall (score 3) was noticed in group 1 and 3 (Clearfil APX composite and one-layer Kalore composite) and the lowest was in group 4 and 5 (one-layer and double-layer P90 composite).

Comparing the scores of dye penetration in the coronal margins revealed no statistically significant difference among the five groups (*p*< 0.423) (Table 2).

**Table 2:** Frequency distribution of dye penetration in coronal margins

**Tested Groups**		**Score of Microleakage**				**Total**	**P value**
		**Score 3**	**Score 2**	**Score 1**	**Score 0**		
Group 1	SE Bond+ Clearfil APX	2(20%)	0	1(10%)	7(70%)	10	0.423
Group 2	GC UniFil Bond +GC Kalore (two layers)	1(10%)	4(40%)	2(20%)	3(30%)	10	
Group 3	GC UniFil Bond +GC Kalore (one layer)	2(20%)	1(10%)	2(20%)	5(50%)	10	
Group 4	P90 adhesive system + Filtek P90 (one layer)	0	0	5(50%)	5(50%)	10	
Group 5	P90 adhesive system + Filtek P90 (two layers)	0	2(20%)	2(20%)	6(60%)	10	
Total		5(10%)	7(14%)	12(24%)	26(52%)	50	

The scores of dye penetration in cervical margins revealed the highest percentage of samples with no dye penetration (score 0) to be in group 4 (one-layer P90), and the lowest percentage in groups 3 and 5 (one-layer Kalore and double-layer P90).Deep dye penetration with the involvement of axial wall (score 3) in cervical margins was most frequently detected in group 3 (one-layer Kalore) and least frequently in group 4 (one-layer P90). The five groups were not significantly different in terms of dye penetration in cervical margin (*p*< 0.212). (Table 3)

**Table 3:** Frequency distribution of dye penetration in cervical margins

**Tested Groups**		**Score of Microleakage**				**Total**	**P value**
		**Score 3**	**Score 2**	**Score 1**	**Score 0**		
Group 1	SE Bond+ Clearfil APX	3(30%)	2(20%)	3(30%)	2(20%)	10	0.212
Group 2	GC UniFil Bond +GC Kalore (two layers)	2(20%)	4(40%)	2(20%)	2(20%)	10	
Group 3	GC UniFil Bond +GC Kalore (one layer)	5(50%)	1(10%)	3(30%)	1(10%)	10	
Group 4	P90 adhesive system + Filtek P90 (one layer)	0	2(20%)	5(50%)	3(30%)	10	
Group 5	P90 adhesive system + Filtek P90 (two layers)	2(20%)	4(40%)	3(30%)	1(10%)	10	
Total		12(24%)	13(26%)	16(32%)	9(18%)	50	

Mann-Whitney test was used to compare the coronal and cervical margin of each group in terms of dye penetration. In this regard, the higher scores were seen in cervical margins; however, significant difference was seen only in group 5 (*p*= 0.018). (Table 4)

**Table 4:** Comparison of dye penetration in cervical margins in dentin versus coronal margins in enamel

**Groups**	**Margin**	**Mean**	**P Value**
Group 1	Cervical	12.75	0.071
	Coronal	8.25	
Group 2	Cervical	11.30	0.526
	Coronal	9.70	
Group 3	Cervical	12.80	0.069
	Coronal	8.20	
Group 4	Cervical	12	0.208
	Coronal	9	
Group 5	Cervical	13.5	0.018
	Coronal	7.5	

## Discussion

Microleakage assessment is among the most common methods of evaluating the quality of dental restoration materials.[3] This article compared the microleakage in a silorane-based composite (Filtek P 90) and two other brands of resin composites, GC Kalore low-shrinkage and conventional Clearfil APX.

Self-etch bonding systems are more hydrophilic compared to total-etch systems.[3] In the current study, in order to eliminate this variation between the two bonding systems and to increase the hydrophilicity of adhesive layers when the samples were exposed to fuchsin solution, only self-etch bonding system was used for all experimental groups.

Compatibility of the adhesive system and the corresponding composite is one of the important clinical features in choosing the materials.[4, 6, 8] Using silorane-based adhesives is essential when employing silorane-containing composites;[3-4,9] however; the monomer used in GC Kalore composite has been reported to be compatible with other adhesives.[4] Nevertheless, in groups with GC Kalore composite, the adhesive used (GC UniFil bond) was among those suggested by the manufacturer.

In this study, prior to application of self-etch bonding, enamel margins were etched by 37% phosphoric acid for 15 seconds because previous studies showed that etching the enamel was efficient in bonding process and increased the marginal adaptation.[10-12]

The null hypothesis of this study was that the new composites with low polymerization shrinkage had no difference in microleakage manifestation compared with the conventional ones. The results supported the hypothesis, as there was no statistically significant difference between the groups in enamel and dentin margins. 

The microleakage scores in coronal/enamel and cervical/ dentin margins in different groups were not significantly different; yet, the least microleakage score was observed in Filtek P90 groups. The findings of this study were in line with those studies that reported the presence of silorane monomer and ring-opening polymerization reaction as the probable major cause of lower microleakage.[2, 6-7]

Other studies reported the silorane-containing composites to have slower initial polymerization reaction.[2, 4, 6, 13-14]After the beginning of the radiation, polymerization process may last up to 20 minutes and this means that silorane circles continue to open until the conformation of three-dimensional polymer network to occur.[6] This factor may help release of stress from polymerization process and decrease the microleakage. Studies done by Gao *et al.*,[15] Al-Boni and Raja,[1] Bagis *et al.*,[16] and Umer *et al.*,[3]showed that Filtek P90 composite had comparable microleakage score with other tested brands of resin composites. These results were in contrast with other studies in which different resin composites were tested.[4-6,8, 17-19] The high viscosity feature of low-shrinkage composites provides better adaptation of this material to cavity walls. Tanno *et al.* studied the silorane-based composites and found larger marginal gaps in some of the samples which were incorporated with bubbles.[7] However, they observed no significant difference between the gaps generated in GC Kalore and those in conventional methacrylate composites.[7]

In the current study, low-shrinkage composite restorations were performed in a bulk/single-layer and also an incremental/double-layer restorative technique, similar to a study by Yamazaki *et al.*[5] The results of some previous studies confirmed that regardless of the restorative system, employing the incremental technique resulted in significantly less microleakage than the bulk technique.[1, 5] On the contrary, Tezvergil- Mutluay *et al.* reported that the use of silorane-containing composite in incremental technique yielded a weaker bond between the layers in comparison with the use of the same technique for conventional resin composite restorations.[20] It might be due to the fact that the polymerization reaction is not hindered superficially with the oxygen inhibitor layers; therefore the bond between the sequential layers in incremental method may not be as strong as the composites containing dimethacrylate.[21] Bagis *et al.*[16] and Yamazaki *et al.*[5] showed that laminal application of this new generation of composite material may reduce the microleakage.

The results of this study presented no statistically significant difference between one layer/bulk and double-layer/incremental techniques. It was also reported that when using silorane-based composite system, the configuration of cavity design and polymerization process via light-curing technique were as effective on bond stability as when dimethacrylate-based composite system was used.[4, 7] Other studies detected that the incremental technique may provide the same quality in deeper cavity designs when the silorane-containing composites were employed.[6, 14] The results of this study do not support the findings reported by Yamazaki *et al.*[5] The main reasons may be the different composites and fatigue testing methods (thermocycling versus load cycling) used in their study.[5] As it was expected and as reported by previous studies,[22-23] the microleakage scores of each group were greater in the cervical margins than the coronal margins. Except in group 5, no significant difference was observed between the enamel/ coronal and dentin/cervical margins.

The finding of this study revealed no statistically significant difference microleakage among all groups. We suggest further studies evaluating the influence of sample storage and load cycling on microleakage. The results would not necessarily translate to clinical practice and future studies in the form of clinical trials are required. 

## Conclusion

Within the limitations of this study, it can be concluded that the microleakage in Filtek P90 and GC Kalore composites were not significantly different from those of the conventional composites. It must be noted that the results of this study can only be reflected to the tested composite materials. 
